# Ten-year follow-up study of a young woman with loeys-dietz syndrome: a case report

**DOI:** 10.1186/s13019-023-02322-1

**Published:** 2023-07-04

**Authors:** Tomohiro Nakajima, Yutaka Iba, Tsuyoshi Shibata, Itaru Hosaka, Jyunji Nakazawa, Nobuyoshi Kawaharada

**Affiliations:** Department of Cardiovascular Surgery, Sapporo Medical University School of Medicine, South-1, West-16, Chuo-ku, Sapporo, 060-8543 Japan

**Keywords:** Loeys-Dietz syndrome, Vasodilation, *TGFBR* mutation, Young

## Abstract

We herein report the 10-year surgical course of a 27-year-old woman who underwent two surgeries after being diagnosed with Loeys-Dietz syndrome. As described in previous cases, this patient developed ectopic arterial enlargement. We followed her temporal changes over a 10-year period, including the changes in computed tomography, pathology, and surgery.

## Background

Loeys-Dietz syndrome (LDS) is an autosomal dominant connective tissue disease characterized by multiple organ complications such as multiple epiphysiopathies, spina bifida, cleft palate, and aortic aneurysms.^1)^ In contrast to connective tissue diseases such as Marfan syndrome, rupture or dissection of arteries without enlargement at a young age has been reported in many cases of LDS. We herein describe a young woman with LDS who had undergone surgery for a 100-mm ascending aortic enlargement at the age of 18 years and subsequently underwent surgery for an enlarged abdominal aortic aneurysm after 10 years of follow-up.^2)^

## Case presentation

**Fig. 1:**
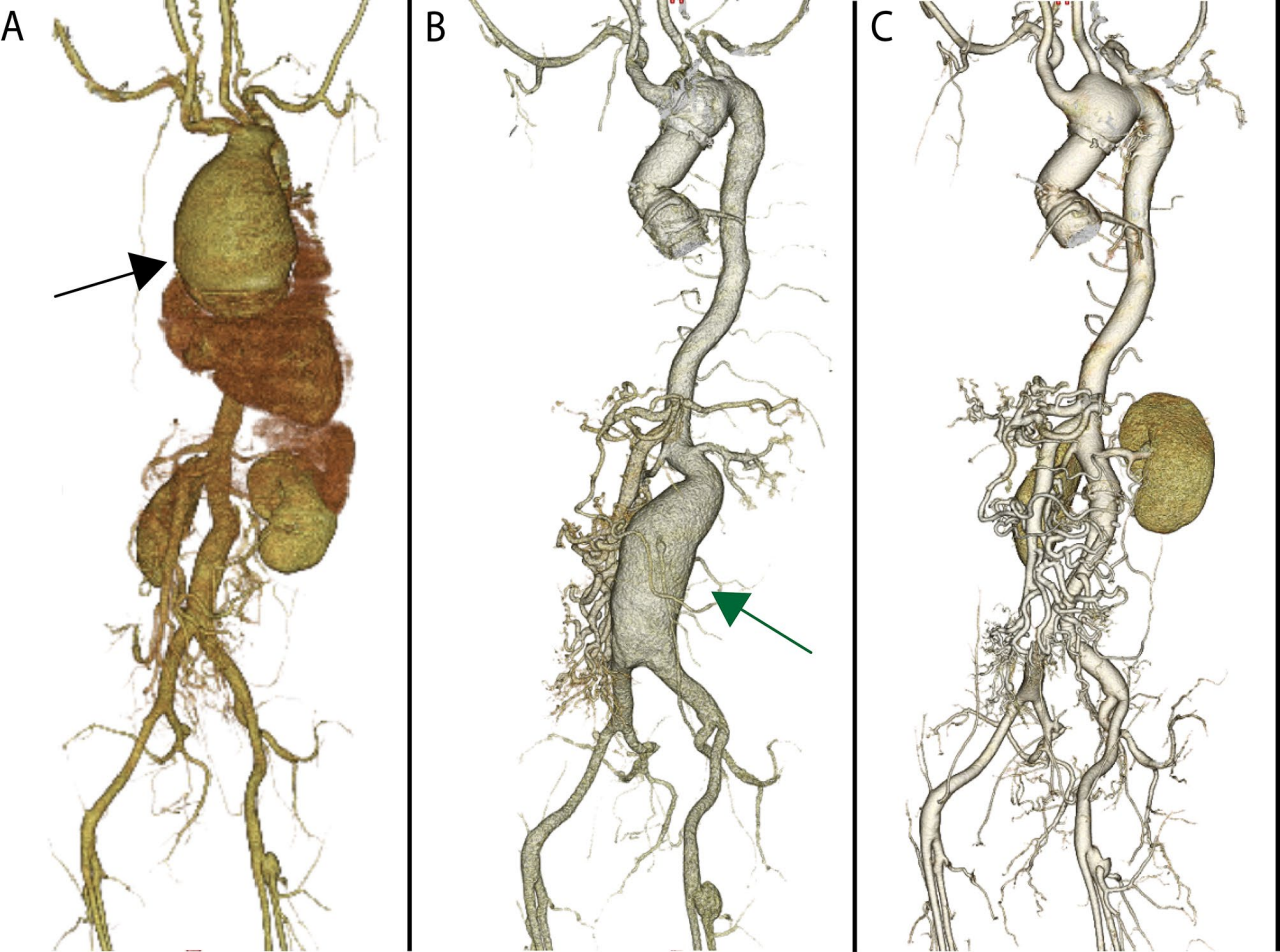
Volume-rendering computed tomography. (**A**) Age of 18 years. Enhanced computed tomography showed 100-mm dilation of the ascending aorta, meandering of the vertebral artery, dilatation of the superior mesenteric artery, and an aneurysm of the left deep femoral artery. (**B**) Age of 27 years. The abdominal aortic artery was dilated. (**C**) Postoperative computed tomography. The abdominal aorta had been replaced by a prosthetic graft, and the aortic arch was dilated

**Fig. 2:**
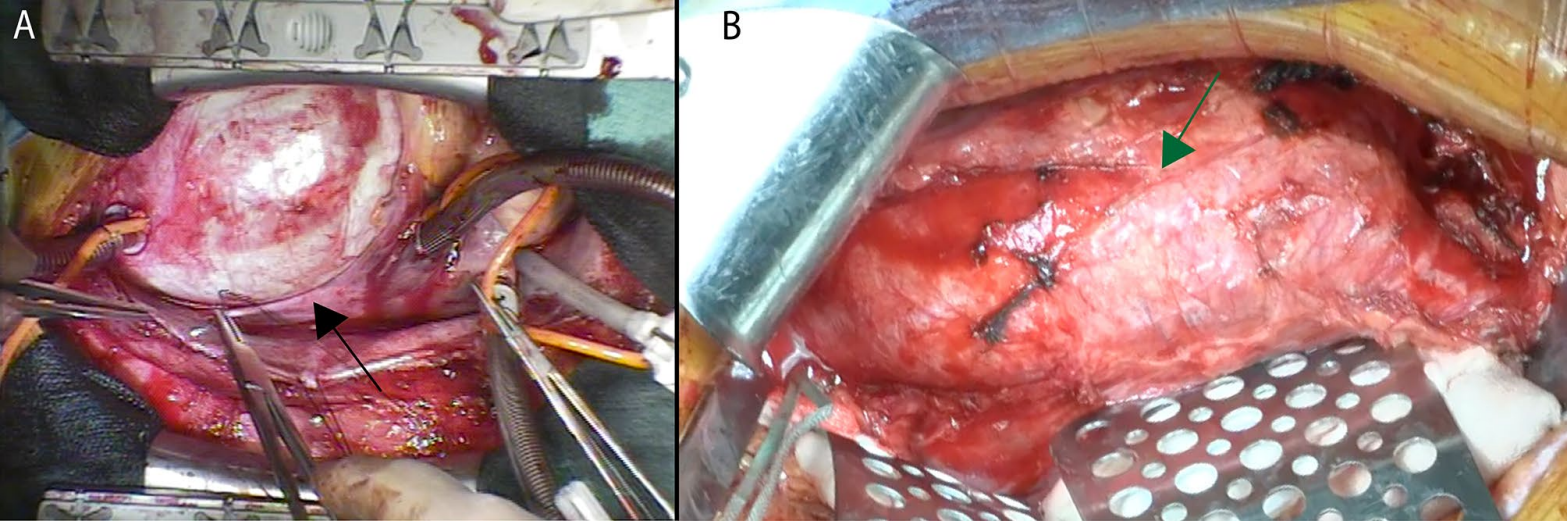
Intraoperative findings. (**A**) At the time of the modified Bentall procedure, the ascending aorta was dilated to 100 mm and white. (**B**) At the time of abdominal aortic graft replacement, the abdominal aorta was dilated and slightly red

A 27-year-old female outpatient at our hospital required surgery for an abdominal aortic aneurysm that had expanded over time (Fig. 1B). At 18 years of age, she had undergone the modified Bentall procedure (Carpentier-Edwards PERIMOUNT Magna Ease valve, 25 mm; Edwards Lifesciences, Inc., Irvine, CA, USA) (Gelweave Valsalva prosthesis, 28-mm; Sulzer Vascutek, Renfrewshire, Scotland) for a 100-mm rapidly enlarging ascending aortic aneurysm and severe aortic regurgitation (Figs. 1A and 2B). Genetic testing revealed a mutation in the *TGFBR1* gene, and the patient was diagnosed with LDS. Her father had died of aortic dissection at 40 years of age. Computed tomography showed meandering of the vertebral artery, 12-mm dilatation of the superior mesenteric artery, 20-mm dilatation of the abdominal aorta, and a 16-mm aneurysm of the left deep femoral artery (Fig. 1A). The patient was followed up as an outpatient, and computed tomography imaging was performed once a year to monitor the arterial enlargement. The abdominal aortic aneurysm enlarged by 2 mm/year for the first 5 years and then by 5 mm/year for the next 5 years, finally reaching 50 mm in 2020. After obtaining the patient’s consent, we performed abdominal aortic artery replacement to prevent rupture. She underwent general anesthesia, and the abdominal aorta was exposed through a midline abdominal incision (Fig. 2B). After general heparinization and an activated coagulation time of > 250 s, the aorta was blocked, the aneurysm was resected, and an artificial blood vessel was replaced (Hemashield, 16 × 9 mm; Maquet, Rastatt, Germany). The inferior mesenteric artery was reconstructed. As shown in Fig. 2B, the abdominal aorta was almost the usual color and thickness, although the ascending aorta was white and thin-walled. Enhanced computed tomography on postoperative day 7 showed the abdominal aorta with the prosthetic graft (Fig. 1C). In addition, the autologous vessels of the aortic arch tended to dilate, requiring careful follow-up of these vessels along with the superior mesenteric artery and left deep femoral artery.

**Fig. 3:**
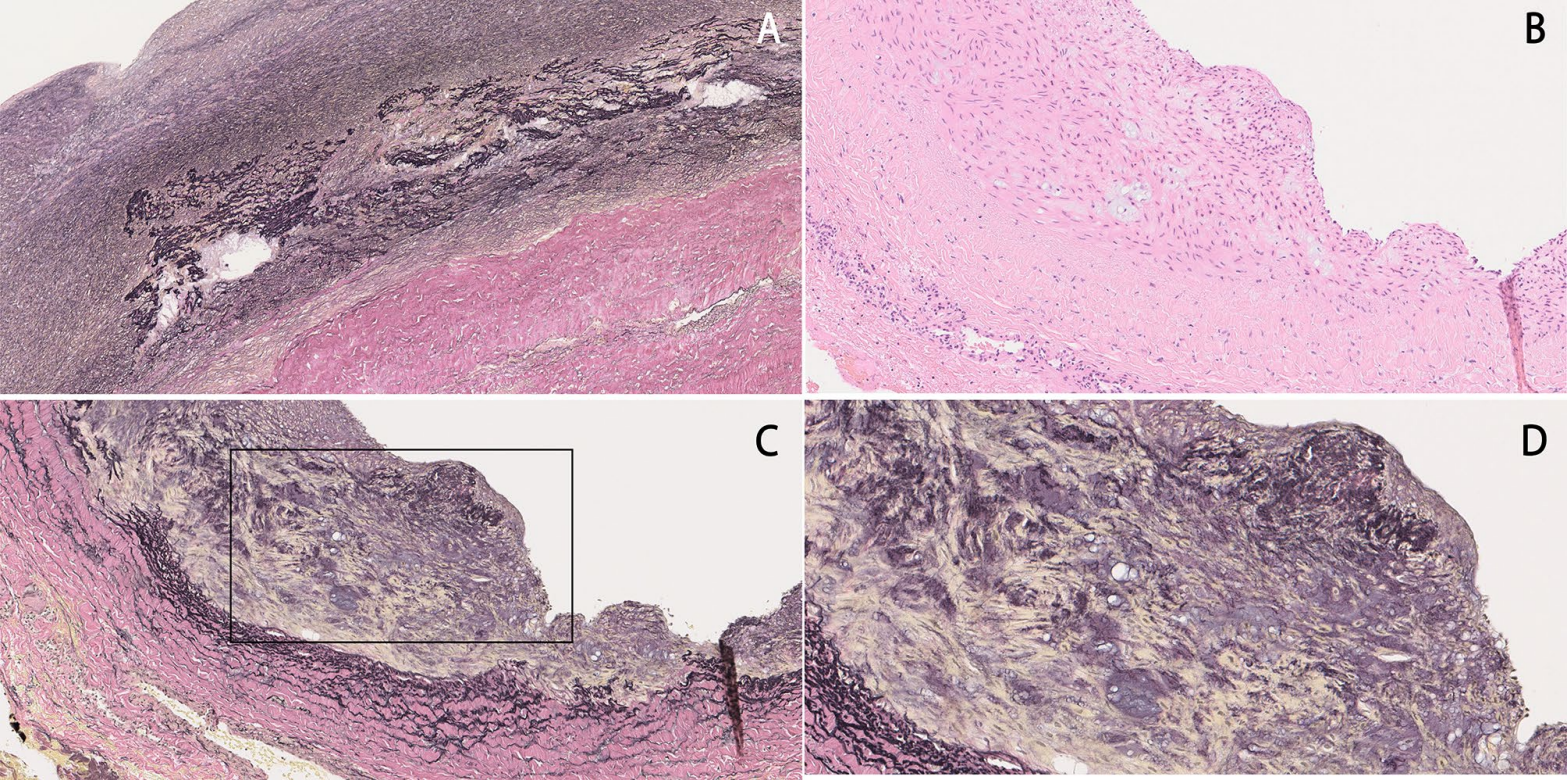
Pathological findings. (**A**) At 18 years of age, elastica van Gieson staining of the ascending aorta showed diffuse degeneration and elastin fragmentation in the media of the ascending aorta. (**B**) Hematoxylin and eosin staining of the abdominal aortic wall showed mucus deposits in the tunica media. (**C**) Elastica van Gieson staining showed loss of tunica media elastic fibers and their cord-like arrangement. (**D**) Higher magnification of Fig. 3C.

Pathologic examination showed no atheroma adherence or calcification, which are characteristic of common aortic aneurysms. At 18 years of age, histopathologic examination of the ascending aortic wall by elastica van Gieson staining had shown diffuse degeneration and elastin fragmentation in the tunica media (Fig. 3A). In the present examination, hematoxylin and eosin staining of the abdominal aortic wall showed mucus deposits in the tunica media (Fig. 3B), and elastica van Gieson staining showed loss of tunica media elastic fibers and their cord-like arrangement (Fig. 3C). Higher magnification is shown in Fig. 3D.

## Discussion and conclusions

LDS is a newly described systemic hereditary connective tissue disease caused by mutations in the *TGFBR* gene.^3)^ It is characterized by cardiovascular, skeletal, cutaneous, and other manifestations, primarily aortic lesions. The clinical presentation ranges from skeletal lesions similar to those of Marfan syndrome and Shprintzen-Goldberg syndrome to hereditary aortic aneurysms alone; however, vascular lesions such as aortic aneurysms, dissections, and arterial tortuosity are present in most cases. At present, genetic analysis is essential for a definitive diagnosis. Although LDS shares many features with related connective tissue diseases, it is not well recognized as a disease; therefore, it is expected that many cases of LDS are misdiagnosed as other diseases, and the number of reported cases is still low worldwide. Therefore, the criteria for selecting effective medical and surgical treatments are also unknown. In terms of disease management and treatment, patients with LDS tend to develop aortic aneurysms and arterial dissection at a younger age than patients with other related diseases; thus, careful cardiovascular management is important from a young age and requires a different response than other related connective tissue diseases such as Marfan syndrome.^4)^

In the present case, histopathologic examination of the aortic wall revealed diffuse degeneration and elastin fragmentation in the tunica media. Because these changes are observed in both LDS and Marfan syndrome, genetic screening is necessary for the diagnosis of LDS.

In our patient, LDS was not diagnosed at the time of the previous surgery. Although controversial, the Bentall procedure using a bioprosthetic valve was performed because of the patient’s strong desire. During the subsequent 10 years, the aortic arch gradually dilated. Given that the patient was 27 years old, it was highly likely that surgery would be required within a few years.^5)^ At the time of this writing, the patient desired to have a baby; however, we have discussed the situation with her and are considering mechanical valve and aortic arch replacement for the next procedure.

Although pathological studies and single case reports of LDS have been published in the past, no case report has described follow-up of the same patient for 10 years. Over the course of the decade in this case, ectopic changes in the aorta were very pronounced and impactful.

In summary, we have herein reported the 10-year surgical course of a young woman diagnosed with LDS. As in previous reports, she developed ectopic arterial enlargement over time. Careful follow-up is needed in future.

## Data Availability

15 July 2022.

## Abbreviations

LDS: Loeys-Dietz syndrome
